# The Role of Growth Retardation in Lasting Effects of Neonatal Dexamethasone Treatment on Hippocampal Synaptic Function

**DOI:** 10.1371/journal.pone.0012806

**Published:** 2010-09-21

**Authors:** Yu-Chen Wang, Chiung-Chun Huang, Kuei-Sen Hsu

**Affiliations:** 1 Department of Pharmacology, College of Medicine, National Cheng Kung University, Tainan, Taiwan; 2 Center for Gene Regulation and Signal Transduction Research, National Cheng Kung University, Tainan, Taiwan; Medical College of Georgia, United States of America

## Abstract

**Background:**

Dexamethasone (DEX), a synthetic glucocorticoid, is commonly used to prevent or lessen the morbidity of chronic lung disease in preterm infants. However, evidence is now increasing that this clinical practice negatively affects somatic growth and may result in long-lasting neurodevelopmental deficits. We therefore hypothesized that supporting normal somatic growth may overcome the lasting adverse effects of neonatal DEX treatment on hippocampal function.

**Methodology/Principal Findings:**

To test this hypothesis, we developed a rat model using a schedule of tapering doses of DEX similar to that used in premature infants and examined whether the lasting influence of neonatal DEX treatment on hippocampal synaptic plasticity and memory performance are correlated with the deficits in somatic growth. We confirmed that neonatal DEX treatment switched the direction of synaptic plasticity in hippocampal CA1 region, favoring low-frequency stimulation- and group I metabotropic glutamate receptor agonist (*S*)-3,5,-dihydroxyphenylglycine**-**induced long-term depression (LTD), and opposing the induction of long-term potentiation (LTP) by high-frequency stimulation in the adolescent period. The effects of DEX on LTP and LTD were correlated with an increase in the autophosphorylation of Ca^2+^/calmodulin-dependent protein kinase II at threonine-286 and a decrease in the protein phosphatase 1 expression. Neonatal DEX treatment resulted in a disruption of memory retention subjected to object recognition task and passive avoidance learning. The adverse effects of neonatal DEX treatment on hippocampal synaptic plasticity and memory performance of the animals from litters culled to 4 pups were significantly less than those for the 8-pup litters. However, there was no significant difference in maternal care between groups.

**Conclusion/Significance:**

Our results demonstrate that growth retardation plays a crucial role in DEX-induced long-lasting influence of hippocampal function. Our findings suggest that therapeutic strategies designed to support normal development and somatic growth may exert beneficial effects to reduce lasting adverse effects following neonatal DEX treatment.

## Introduction

The synthetic glucocorticoid dexamethasone (DEX) is commonly used to prevent or treat chronic lung disease in preterm infants with respiratory distress syndrome [1], [2], [3]. The treatment regimen typically consists of high doses of DEX for several weeks, notably during a critical period for the development of brain function. Therefore, growing concern has arisen for the long-term safety of this clinical practice on the brain development of the child. There is now increasing evidence that early-life DEX exposure negatively affects somatic growth and is associated with an increased risk of adverse neurodevelopmental outcome. Although long-term follow-up studies are scarce as yet, a recent study reported that school-age children who were treated with DEX as preterm infants have impaired neuromotor skills and cognitive performance [4]. Barrington's review of 8 randomized controlled studies enrolling 679 infants also showed a significant risk for neurodevelopmental abnormalities and cerebral palsy after early DEX treatment [5]. These findings raise important questions concerning the mechanisms underlying these neurodevelopmental abnormalities and whether these alterations are long-lasting. Given controlled prospective and mechanistic study with humans is limited, a convenient way to answer these questions is using an appropriate animal model that simulates the DEX treatment used clinically during neonatal life.

To date, a number of clinically relevant animal models have been developed to investigate the lasting neurological outcome of neonatal DEX treatment. It is estimated that in terms of brain growth rate, the brain of a rat pup around birth is comparable with that of a preterm human infant brain, born during 26–32 weeks gestation [6], [7]. Given these developmental correlations, a 3-day tapering course of DEX treatment in the neonatal rat pup on postnatal days 1–3 (P1-3) has been developed to investigate lasting effects of neonatal DEX treatment on the developing brain [8], [9], [10]. With the use of this model, we and others have recently demonstrated that DEX exposure in the neonatal rat pups may lead to alterations in synaptic plasticity and memory performance in the adult hippocampus [8], [9]. Although these results highlight the risk for long-lasting behavioral and physiological alterations associated with neonatal DEX treatment, little is known about the mechanisms behind these abnormalities. As a result, there is at present no comprehensive strategy to combat adverse effects of neonatal DEX treatment on neurodevelopment.

Clinical studies have demonstrated that preterm infants receiving DEX therapy have reduced growth rate, decreased weight gain, and smaller head circumferences [11], [12]. There is also ample evidence in animal studies that neonatal DEX treatment causes long-lasting adverse effects on gross somatic growth and brain weight [9], [13], [14]. The causal link between the decreased somatic growth and the occurrence of abnormal neurological outcomes induced by neonatal DEX treatment has not yet been established. Interestingly, we have recently demonstrated that although DEX exposure in the neonatal rat pups alters hippocampal synaptic plasticity and the formation of contextual fear memory in adolescence, these effects do not persist into adulthood [9]. This motivated us to investigate whether supporting normal somatic growth may overcome the adverse effects of neonatal DEX treatment on hippocampal function in later life. Because the litter size in which rodent pups are reared is known to affect their preweaning somatic growth [15], [16], we employ culling (a reduction in the litter size) advantage for measuring the role of growth retardation in lasting effects of neonatal DEX treatment on hippocampal synaptic plasticity and function.

## Results

### Effect of culling on somatic growth retardation induced by neonatal DEX treatment

**Figure 1:**
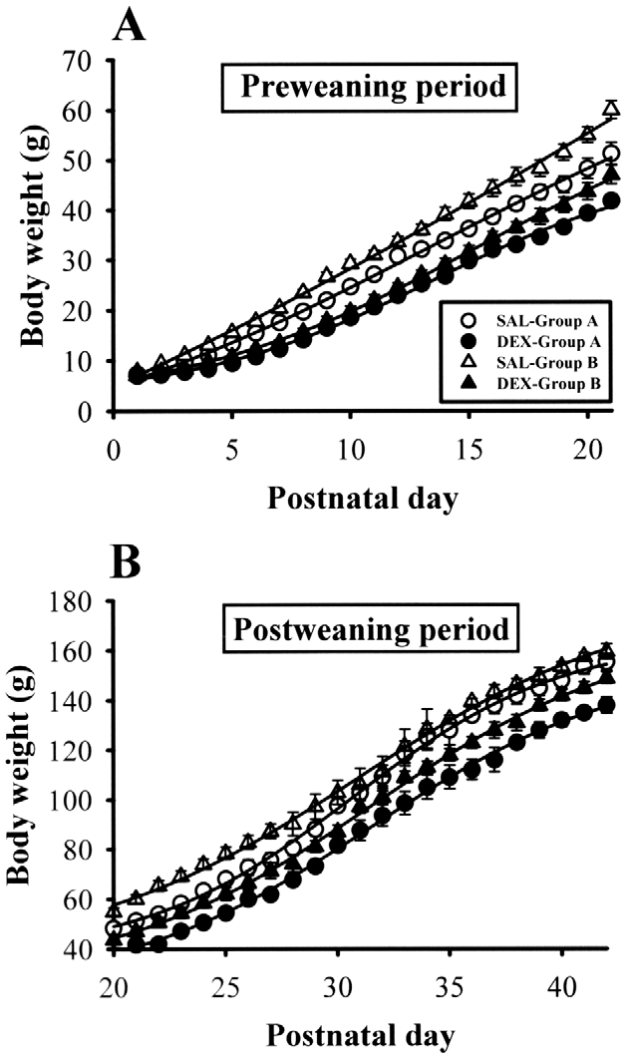
Effect of neonatal DEX treatment on body weight. (A) DEX-treated rats had significantly lower body weights compared with SAL-treated rats at most ages during the preweaning period (P1-P21) for Group A (8 pups/litter) and Group B (4 pups/litter). (B) DEX-treated rats weighted significantly less than SAL-treated rats during the postweaning period (P22-P42) for Group A and Group B. Data represent the mean ± SEM (n = 10 for each group).

To study the role of growth retardation in lasting adverse effects of neonatal DEX treatment on hippocampal synaptic plasticity and function, we took advantage of culling to increase body weight gain [15], [16]. We first examined the influence of litter size on postnatal somatic growth of rat pups. In agreement with previous observations [8], [9], [13], [14], neonatal DEX-treated pups gained weight at a slower rate than saline (SAL)-treated rats for up to 6 weeks after treatment was discontinued (Figure 1). Compared to SAL-treated rat pups from litters culled to 8 (Group A), the rat pups from litters culled to 4 (Group B) showed a faster growth rate on postnatal (P)8-P28 (p<0.05). By P29-P42, there were no significant differences in body weight between SAL-treated Group A and Group B. In addition, post hoc analysis revealed that DEX-treated rat pups in Group B weighed significantly higher than those in DEX-treated rat pups in Group A on P17-P42 (p<0.05), but by P1-P16 there was no body weight difference between groups. These results suggest that culling can partially, but significantly, overcome the growth retardation induced by neonatal DEX treatment.

In contrast to the adverse effect on somatic growth, histopathological evaluation using cresyl violet staining showed that neonatal DEX treatment does not significantly affect the total number of neurons in the stratum pyramidale of the hippocampal CA1 region compared with SAL-treated rats at 5 weeks of age (p>0.05, unpaired Student's *t*-test) (Figure S1).

### Effect of culling on maternal care

**Figure 2:**
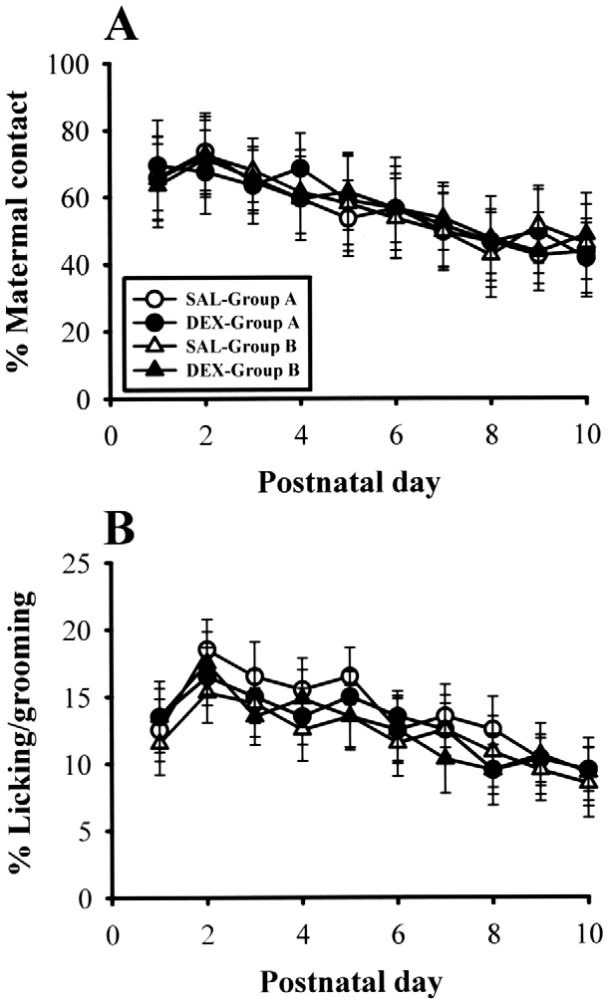
Effect of litter size on maternal care over the first 10 days postpartum. (A) Percentage of time spent in maternal contact (the total of all behavioral categories involving contact between dam and pups including licking/grooming, arched-back nursing, flat-back nursing, passive nursing) across days in neonatal SAL- and DEX-treated rat pups for Group A and Group B. (B) Percentage of time spent in maternal licking/grooming behavior across days in neonatal SAL- and DEX-treated rat pups for Group A and B. Data represent the mean ± SEM (n = 8 for each group).

The quality of postpartum maternal care may modulate hippocampal development and function [17]. To determine whether culling may influence maternal care of pups, we measured the amount of time spent in contact between dam and pups over the first 10 days postpartum. Maternal contact was defined as any behavior that involved in physical contact or close proximity to pups and almost invariably implied nursing and/or licking/grooming [18], [19]. As shown in Figure 2A, the percentage of time spent in the maternal contact during the observation period did not differ between neonatal SAL- and DEX-treated rat pups in both Group A and Group B. Further statistical analysis of data revealed that the frequency of maternal licking/grooming was not different between groups across all days of the observation period (Figure 2B). In addition, as reported previously [20], no indications were found for altered maternal care received by neonatal SAL- and DEX-treated rat pups.

### Effect of culling on neonatal DEX treatment-induced alteration in glutamatergic synaptic transmission

**Figure 3:**
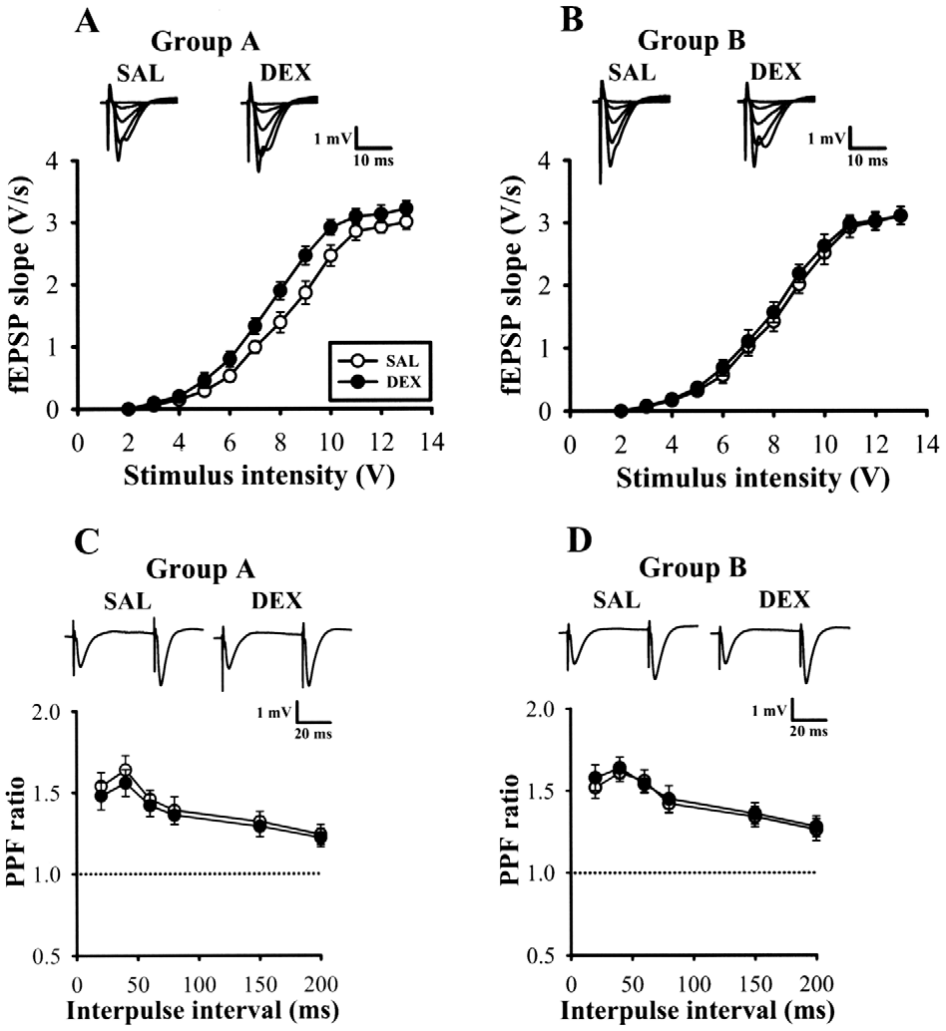
Culling reduces the effects of neonatal DEX treatment on basal synaptic transmission in hippocampal CA1 region. (A) Stimulus-response curve of fEPSP versus stimulus intensity at the Schaffer collateral-CA1 synapses of hippocampal slices from 5-week-old rats of Group A that received neonatal SAL (n = 5) or DEX treatment (n = 5). Inset shows overlaid traces (each an average of 3 responses) evoked in a slice from a SAL- (left set of traces) and DEX-treated (right set of traces) rats. (B) Stimulus-response curve of fEPSP versus stimulus intensity at the Schaffer collateral-CA1 synapses of hippocampal slices from 5-week-old rats of Group B that received neonatal SAL (n = 5) or DEX treatment (n = 5). (C) Comparison of PPF ratio in slices from 5-week-old rats of Group A that received neonatal SAL (n = 5) or DEX treatment (n = 5). The plot summarizes facilitation of the second fEPSP slope relative to the first one as a function of the interpulse intervals of 20–200 ms. The inset shows example PPF (average of 3 responses) obtained with interpulse interval of 40 ms in slices from SAL- and DEX-treated rats. (D) Comparison of PPF ratio in slices from 5-week-old rats of Group B that received neonatal SAL (n = 5) or DEX treatment (n = 5). Data represent the mean ± SEM.

An increased glutamatergic synaptic transmission at the Schaffer/collateral-CA1 synapses following neonatal DEX treatment has been reported previously [9]. Therefore, in this experiment, we examined the influence of culling on the effect of neonatal DEX treatment on basal glutamatergic synaptic transmission by comparing stimulus-response relationships for extracellular field excitatory postsynaptic potential (fEPSP). As compared with slices from SAL-treated 5-week-old rats, neonatal DEX-treated rats in Group A showed a significant leftward shift in the stimulus-response curve of the fEPSP (p<0.05) (Figure 3A). However, no significant differences in stimulus-response relationships were found between SAL- and DEX-treated rats in Group B (p>0.05) (Figure 3B). These findings are in agreement with previous report [9] and support the view that neonatal DEX treatment enhances basal glutamatergic synaptic transmission in the hippocampal CA1 region in later life and such effect is effectively overcome by culling.

We next tested whether the effect of neonatal DEX treatment on basal synaptic transmission reported above was attributable to an increase in presynaptic probability of neurotransmitter release. To address this question, we examined the paired-pulse facilitation (PPF), a short-lasting form of presynaptic plasticity in which the second of two closely spaced stimuli elicits enhanced transmitter release [21]. As shown in Figure 3C and 3D, pairs of presynaptic fiber stimulation pulses delivered at interpulse intervals of 20, 40, 60, 80, 150, and 200 ms evoked nearly identical amounts of PPF ratio in slices from DEX- and SAL-treated rats from both Group A and Group B. These results suggest that the presynaptic function at the Schaffer/collateral-CA1 synapses remains normal following neonatal DEX treatment.

### Effect of culling on neonatal DEX treatment-induced alterations in the induction of long-term potentiation and long-term depression

**Figure 4:**
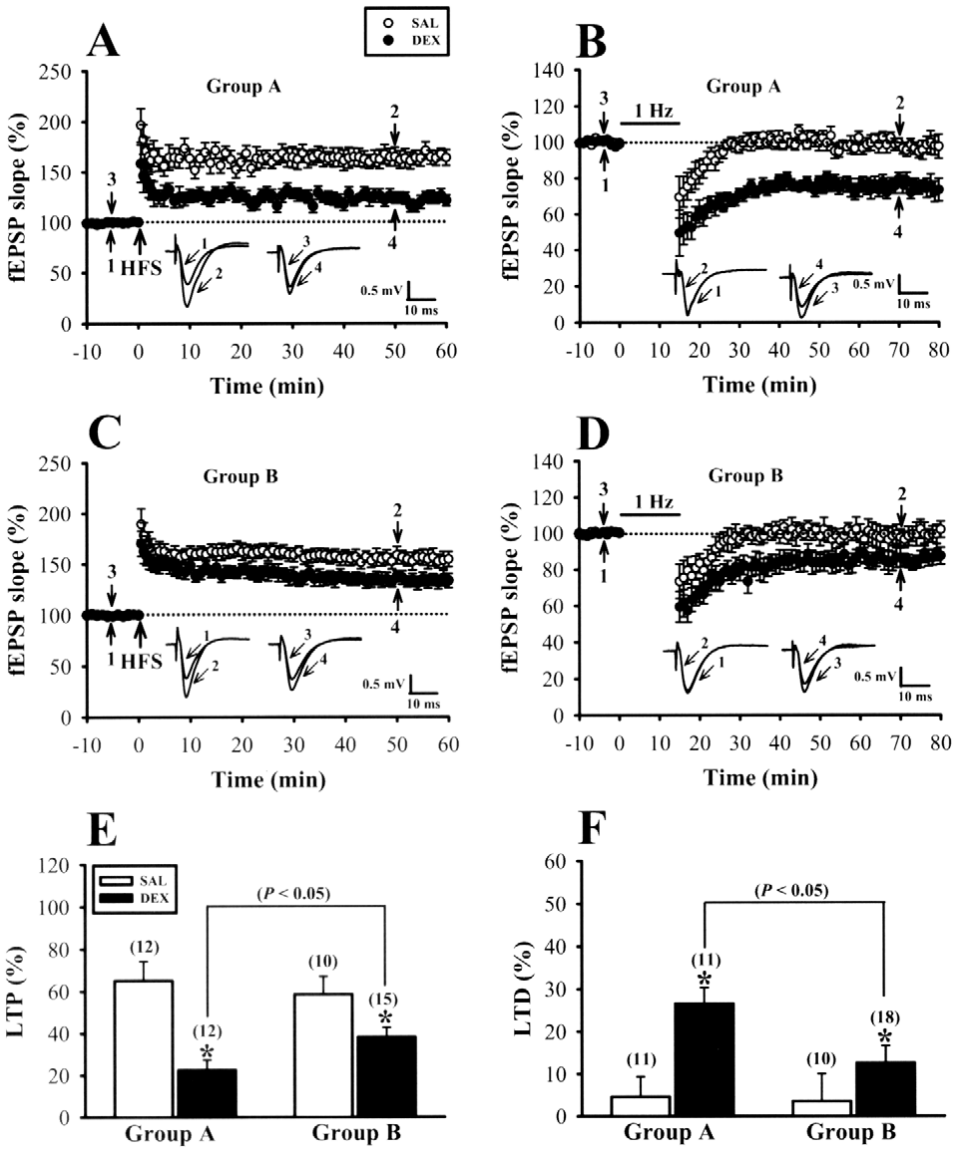
Culling reduces the effects of neonatal DEX treatment on LTP and LTD in hippocampal CA1 region. (A) Summary graphs of LTP induced with a HFS in slices from 5-week-old rats of Group A that received neonatal SAL or DEX treatment. (B) Summary graphs of LTD induced with a prolonged LFS in slices from 5-week-old rats of Group A that received neonatal SAL or DEX treatment. (C) Summary graphs of LTP induced with a HFS in slices from 5-week-old rats of Group B that received neonatal SAL or DEX treatment. (D) Summary graphs of LTD induced with a prolonged LFS in slices from 5-week-old rats of Group B that received neonatal SAL or DEX treatment. (E) Summary bar graphs depicting levels of potentiation measured 50 min after HFS in slices from neonatal SAL- or DEX-treated rats for Group A and Group B. (F) Summary bar graphs depicting levels of depression measured 50 min after LFS in slices from neonatal SAL- or DEX-treated rats for Group A and Group B. The total number of animals examined is indicated by n in parenthesis. Representative traces of fEPSPs were taken at the time indicated by number. Horizontal bars denote the period of delivery of LFS. The dashed lines show the level of baseline. Data represent the mean ± SEM. *p<0.05 compared with SAL-treated rats.

Previous studies from our laboratory and those of others have demonstrated that tapering neonatal DEX treatment may alter the inducibility of hippocampal CA1 long-term potentiation (LTP) and long-term depression (LTD) in later life [8], [9], [10]. To determine the influence of culling on the effects of neonatal DEX treatment on long-term synaptic plasticity, we analyzed the induction of LTP and LTD in the CA1 region of the hippocampus. In slices from 5-week-old SAL-treated rats, conditioning high-frequency stimulation (HFS) induced a robust LTP of fEPSPs (50 min after HFS: Group A, 165.1±9.1% of baseline, n = 12, p<0.05; Group B, 158.6±8.5% of baseline, n = 10, p<0.05) (Figures 4A, 4C and 4E). There was a striking difference in the effect of neonatal DEX treatment on LTP induction between Group A and Group B. Although neonatal DEX treatment resulted in an impaired LTP induction in slices from both Group A (122.5±4.8% of baseline, n = 12) and Group B rats (138.2±4.6% of baseline, n = 15), the impairment of LTP in slices from Group B rats was significantly less than those from Group A rats (p<0.05) (Figure 4E). On the contrary, LTD was induced by applying low-frequency stimulation (LFS) at 1 Hz for 15 min. As shown in Figures 4B and 4D, slices from 5-week-old DEX-treated rats showed a reliable LFS-induced LTD (50 min after the end of LFS: Group A, 74.5±4.2% of baseline, n = 11, p<0.05; Group B, 86.3±4.5% of baseline, n = 18, p<0.05) compared with slices from age-matched SAL-treated rats (Group A, 95.4±4.8% of baseline, n = 11; Group B, 96.5±6.5% of baseline, n = 10). However, the magnitude of LFS-induced LTD in neonatal DEX-treated rats was significant reduced in slices from Group B rats compared with those from Group A rats (p<0.05) (Figure 4F).

**Figure 5:**
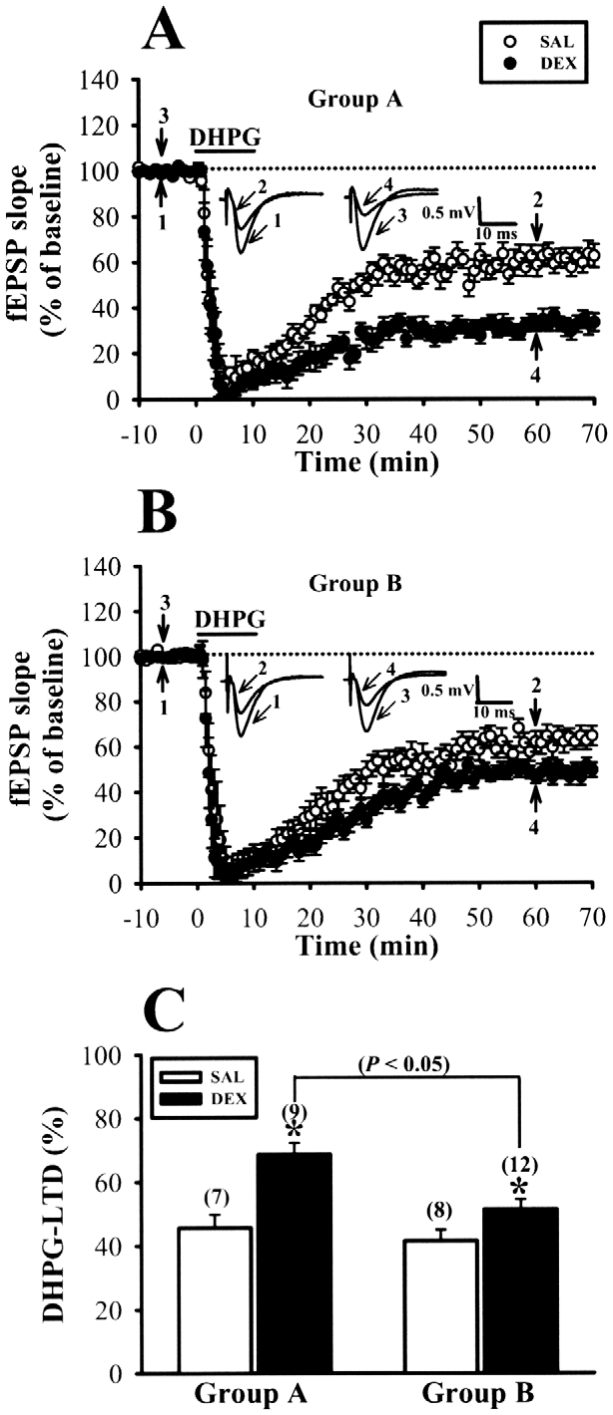
Culling reduces the facilitatory effect of neonatal DEX treatment on DHPG-induced LTD in hippocampal CA1 region. (A) Summary graphs of LTD induced with a brief application of mGluR agonist DHPG (50 µM) for 10 min in slices from 5-week-old rats of Group A that received neonatal SAL or DEX treatment. (B) Summary graphs of LTD induced with a brief application of DHPG (50 µM) for 10 min in slices from 5-week-old rats of Group B that received neonatal SAL or DEX treatment. (C) Summary bar graphs depicting levels of depression measured 50 min after washout of DHPG in slices from neonatal SAL- or DEX-treated rats for Group A and Group B. The total number of animals examined is indicated by *n* in parenthesis. Representative traces of fEPSPs were taken at the time indicated by number. Horizontal bars denote the period of delivery of DHPG. The dashed lines show the level of baseline. Data represent the mean ± SEM. *p<0.05 compared with SAL-treated rats.

At the Schaffer collateral-CA1 synapses, N-methyl-D-aspartate (NMDA) receptor-dependent LTD coexists with another form of LTD mediated by activation of group I metabotropic glutamate receptors (mGluRs) [22]. This mGluR-dependent LTD can be elicited by bath application of a group I mGluR agonist (S)-3,5,-dihydroxyphenylglycine (DHPG) over a short period of time [23], [24], [25]. Moreover, these two forms of LTD differ with respect to their underlying mechanisms [26], [27]. We then examined whether the induction of mGluR-dependent LTD was also altered by neonatal DEX treatment. In slices from 5-week-old SAL-treated rats, a brief bath application of DHPG (50 µM) for 5 min elicited a robust LTD (50 min after washout of DHPG: Group A, 54.4±4.2% of baseline, n = 7, p<0.05; Group B, 58.5±3.5% of baseline, n = 8, p<0.05) (Figures 5A and 5B). Slices from neonatal DEX-treated rats displayed an enhanced DHPG-induction LTD (Group A, 31.3±3.6% of baseline, n = 9; Group B, 49.4±3.6% of baseline, n = 12) compared with slices from SAL-treated rats. Likewise, the magnitude of DHPG-induced LTD in neonatal DEX-treated rats was significant reduced in slices from Group B rats compared with slices from Group A rats (p<0.05) (Figure 5C). Overall, these results suggest that neonatal DEX treatment impairs LTP but enhances LTD induction in the hippocampal CA1 region in later life and such effects are effectively overcome by culling.

**Figure 6:**
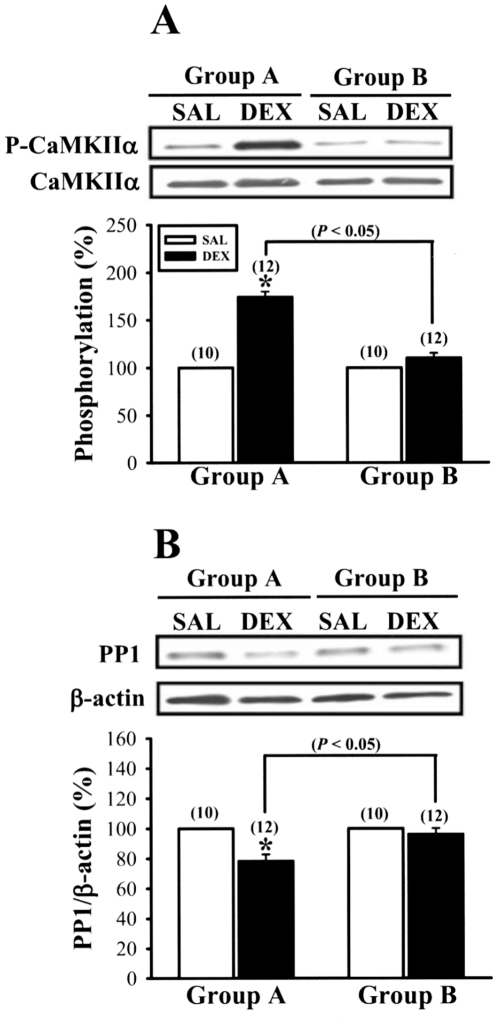
Culling reduces the effects of neonatal DEX treatment on CaMKIIα phosphorylation at threonine-286 and PP1 expression in hippocampal CA1 region. (A) Representative Western blot and summary bar graph depicting levels of CaMKIIα phosphorylation at threonine-286 in hippocampal CA1 PSD purified from 5-week-old rats neonatally treated with SAL or DEX for Group A and Group B. (B) Representative Western blot and summary bar graph depicting levels of PP1 in hippocampal CA1 homogenates from 5-week-old rats neonatally treated with SAL or DEX for Group A and Group B. The total number of animals examined is indicated by n in parenthesis. Data represent the mean ± SEM. *p<0.05 compared with SAL-treated rats.

In previous studies [9], [10], we established that the effects of neonatal DEX treatment on LTP and LTD induction are correlated with an increase in the autophosphorylation of α isoform of Ca^2+^/calmodulin-dependent protein kinase II (CaMKIIα) at threonine-286 in hippocampal postsynaptic density (PSD) fraction and a decrease in the protein phosphatase 1 (PP1) expression in hippocampal CA1 homogenate. Accordingly, we examined the influence of culling on the effects of neonatal DEX treatment on the basal levels of phosphorylated CaMKIIα at threonine-286 and PP1 protein expression. As previous shown [9], in Group A, neonatal DEX treatment led to an increase in CaMKIIα autophosphorylation (174.1±5.8%, n = 12, p<0.05) (Figure 6A) and a decrease in PP1 expression (78.2±4.6%, n = 12, p<0.05) compared with neonatal SAL treatment (Figure 6B). However, no significant effects of neonatal DEX treatment on the levels of CaMKIIα autophosphorylation (110.2±5.2%, n = 12, p>0.05) and PP1 protein (96.1±4.1%, n = 12, p>0.05) were seen in rats from Group B compared with SAL-treated rats (Figures 6A and 6B).

Many cellular stimuli that elevate intracellular free Ca^2+^ may increase CaMKIIα autophosphorylation, including an increase in intrinsic neuronal excitability. To test this possibility, we measured hippocampal CA1 neuronal excitability by quantifying the number of action potentials fired in response to postsynaptic depolarizing current injection (0.2 nA, 500 ms). As shown in Figure S2, there was no significant difference between slices from SAL- and DEX-treated rats in the firing responses to depolarizing current injections. We also found no significant changes on membrane potential, action potential threshold, or kinetics in hippocampal CA1 pyramidal neurons in slice from DEX-treated rats compared with SAL-treated rats at 5 weeks old.

Given that GABAergic disinhibition may serve as a mechanism for generalized enhancement of neuronal excitability and parvalbumin-expressing interneurons have been reported to play a key role in coordinating of neuronal network excitability of the hippocampus [28], we then examined whether neonatal DEX treatment affects parvalbumin-expressing interneurons. Morphological studies have revealed that at least two types of parvalbumin-expressing interneurons in the hippocampus: large, multiple-polar round shaped, axon-somal basket cells and smaller, spindle shaped, axo-axonic chandelier cells [28]. Quantitative analysis indicated that there was no significant effect of neonatal DEX treatment on the total number of multiple-polar round shaped and spindle shaped parvalbumin-expressing interneurons in the stratum origin and pyramidale of the hippocampal CA1 region in rats from both Group A and Group B (Figure S3). In addition, culling resulted in no significant differences in the total number of parvalbumin-expressing interneurons.

To further assess whether neonatal DEX treatment leads to a change in the GABA_A_ receptor-mediated inhibition, we compared inhibitory postsynaptic currents (IPSCs) recorded in hippocampal CA1 pyramidal neurons in slices from SAL- and DEX-treated rats. Monosynaptic IPSCs were evoked while holding neurons in voltage-clamp at −20 mV in the presence of 6-cyano-7-nitroquinoxaline-2,3-dione (CNQX; 20 µM) and D-(-)-2-amino-5-phosphonopentanoic acid (D-APV; 50 µM). Figure S4 depicts the relationship between stimulus intensity and IPSC amplitude. No significant differences in stimulus-response relationships were found between SAL- and DEX-treated rats of both Group A and Group B (Figure S4A and S4B). We also examined the effect of neonatal DEX treatment on miniature IPSC (mIPSC) properties in hippocampal CA1 neurons. Neonatal DEX treatment did not significantly affect the mean frequency and amplitude of mIPSCs compared with SAL-treated rats in both Group A and Group B (Figure S4C and S4D).

### Effect of culling on neonatal DEX treatment-induced impairment of hippocampus-dependent associative learning and memory

**Figure 7:**
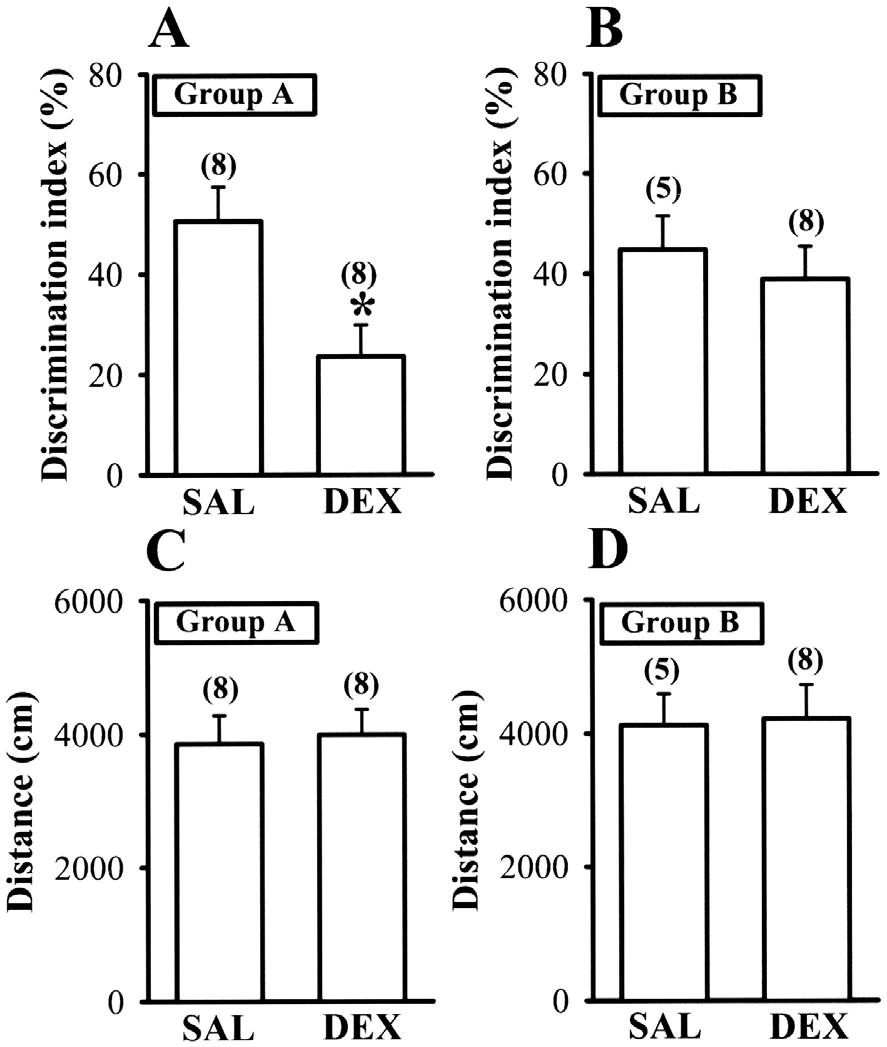
Culling rescues object recognition memory deficits in neonatal DEX rats. (A) Performance in the object recognition task is expressed as a discrimination index corresponding to the percentage of increase above the time spent exploring the familiar object during the test session. Summary bar graphs depicting levels of discrimination index from 5-week-old rats neonatally treated with SAL or DEX for Group A. (B) Summary bar graphs depicting levels of discrimination index from 5-week-old rats neonatally treated with SAL or DEX for Group B. (C) Summary bar graphs depicting total distance traveled during a 5 min trial of 5-week-old rats neonatally treated with SAL or DEX for Group A. (D) Summary bar graphs depicting total distance traveled during a 5 min trial of 5-week-old rats neonatally treated with SAL or DEX for Group B. The total number of animals examined is indicated by n in parenthesis. Data represent the mean ± SEM. *p<0.05 compared with SAL-treated rats.

We have previously found that neonatal DEX treatment may impair hippocampus-dependent learning and memory task [9], [10]. We next investigated whether culling could also reduce learning and memory deficits of neonatal DEX-treated rats measured in the object recognition and passive avoidance tests. At 5 weeks of age, neonatal DEX-treated rats in Group A showed significant decline in the recognition of a novel object [F_(1,15)_ = 8.5, p = 0.01], observed as a decreased time of exploring or sniffing the novel object (Figure 7A). However, neonatal DEX-treated rats in Group B performed significantly better on the object recognition task than did neonatal DEX-treated rats in Group A [F_(1,15)_ = 4.3, p = 0.04]. Notably, the object recognition index did not significantly differ between neonatal SAL- and DEX-treated rats in Group B [F_(1,12)_ = 0.4, p = 0.56] (Figure 7B). The total distance traveled did not differ between groups during the memory retention tests (Figures 7C and 7D).

**Figure 8:**
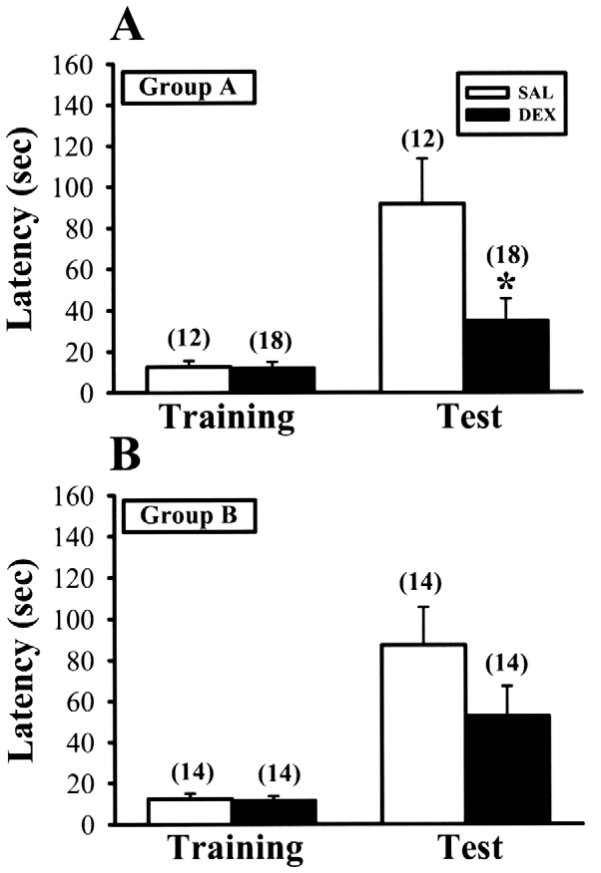
Culling rescues neonatal DEX treatment-induced impairment of memory retention for one-trial passive avoidance task at 5 weeks old. (A) Retention performance in one-trial passive avoidance task is expressed the latency time required for the animal to enter the dark compartment. Summary bar graphs depicting the latencies to enter the dark compartment on retention testing session in 5-week-old rats neonatally treated with SAL or DEX for Group A. (B) Summary bar graphs depicting the latencies to enter the dark compartment on retention testing session in 5-week-old rats neonatally treated with SAL or DEX for Group B. The total number of animals examined is indicated by n in parenthesis. Data represent the mean ± SEM. *p<0.05 compared with SAL-treated rats.

In one-trial passive avoidance test, no significant between-group differences in performance were noted during the training session. But on the day of testing, performance of 5-week-old neonatal DEX-treated rats in Group A have significantly shorter step-through latencies to enter the dark compartment [34.8±10.7 seconds, n = 18; F_(1,29)_ = 6.5, p = 0.02] than SAL-treated rats (91.5±22.1 seconds, n = 12) (Figure 8A). In contrast, there was no significant difference in memory retention performance between neonatal SAL- (87.4±18.4 seconds, n = 14) and DEX-treated rats [52.6±14.6 seconds, n = 14; F_(1,27)_ = 2.2, p = 0.15] in Group B at 5 week old (Figure 8B). These results clearly show that hippocampus-dependent associative learning and memory is impaired by neonatal DEX treatment and culling can effectively overcome these memory impairments.

## Discussion

The present study investigated the role of growth retardation in lasting detrimental effects of neonatal DEX treatment on hippocampal synaptic plasticity and memory performance. Our results confirmed that neonatal DEX treatment retarded somatic growth and impaired LTP but enhanced LTD in hippocampal CA1 region in later life [8], [9], [10]. In addition, neonatal DEX-treated rats showed impaired memory performance in the tasks of object recognition and passive avoidance conditioning. Increasing preweaning milk intake by culling significantly stimulated somatic growth and overcame the adverse effects of neonatal DEX treatment on hippocampal synaptic plasticity and memory performance. Improved performance in hippocampus-associated learning and memory tasks and synaptic plasticity by culling was not associated with any detectable alteration in postpartum maternal care or total number of pyramidal neurons or parvalbumin-expressing interneurons in the CA1 region of the hippocampus.

Normal somatic growth is the result of the proper interactions of genetic, nutritional, metabolic, and endocrine factors. Both clinical and experimental studies have shown that early-life DEX exposure has a lasting adverse effect on gross somatic growth [9], [11], [12], [13], [14]. Consistent with these earlier findings, our results indicated that DEX-treated rats weighted significantly less than those of SAL-treated rats up to 6 weeks after cessation of the treatment (Figure 1). Although the cause of the lasting growth retardation observed in DEX-treated rats remain unclear, this adverse effect may be linked, at least in part, to the inadequate nutritional intake during the postnatal period [3] or the rise in tissue catabolism or protein breakdown after DEX treatment [14], [29]. Because we and others did not find the difference in maternal care between SAL- and DEX-treated groups [20], the somatic growth deficits observed in DEX-treated rats cannot be explained by the deleterious effects of child abuse or neglect. Although several previous studies have reported that brain weights are decreased after neonatal DEX treatment [13], [14], [30] and that neuronal progenitor cells in the dentate gyrus are subjects to DEX-induced apoptosis [31], we found no significant effect of neonatal DEX treatment on the total number of pyramidal neurons or parvalbumin-expressing interneurons in hippocampal CA1 region (Figures S1 and S3). These findings are not consistent with those of Kreider et al [32], who reported that neonatal DEX treatment elicited small but relatively consistent deficits in the number and size of neural cells in the hippocampus that persisted into adulthood. The reason for this discrepancy is unclear, but it could be attributed to the use of different regimens and doses of DEX (daily doses of 0.05, 0.2 or 0.8 mg/kg versus tapering doses of 0.5 mg/kg on P1, 0.3 mg/kg on P2, and 0.1 mg/kg on P3 in our study), resulting in activation of different cellular processes that vary in their mode of action. Another important difference is that Kreider et al [32] measured cell number and size by the DNA concentration and content while we used immunohistochemical staining method in our experiments. Much more work, however, is necessary to fully understand this issue.

We and others have previously reported that neonatal DEX treatment alters hippocampal synaptic plasticity and function in later life [8], [9], [10]. Our data also identified a close link between somatic growth impairment and long-lasting deficits in hippocampal synaptic function in neonatal DEX-treated rats [9]. At 8-week-old age, at which no significant differences in body weight were observed between neonatal SAL- and DEX-treated rats, DEX-treated rats exhibited normal hippocampal synaptic plasticity and contextual fear memory performance compared with SAL-treated rats [9]. In the present study, we have, in addition, extended these findings by demonstrating that culling can support somatic growth and effectively overcome the adverse effects of neonatal DEX treatment on hippocampal LTP and LTD, as well as hippocampus-dependent associative learning and memory performance. Furthermore, we show that the heightened basal synaptic transmission observed in slices from neonatal DEX-treated rats was also overcome by culling. In rodents, litter size affects pup postnatal growth and development [15], [16]. The culling paradigm is one of the few known experimental processes that can accelerate somatic growth in preweaning period of rat pups. The question then arises as to how litter size affects the postnatal growth of rat pups. The correlation between the body weight gain of pups and the litter size is generally believed to be due to the availability of maternal milk [33], [34]. That is pups from culled litters may be heavier due to greater milk availability. Furthermore, we found that maternal contact did not become higher as litter size culled to 4 (Figure 2), suggesting that the influence of litter size on the postnatal growth of rat pups was not associated with alterations in postpartum maternal care. This is in agreement with Champagne et al [19] who reported that litter size has little effect on interaction between mother and pups. However, we could not exclude the possibility that factors other than increased milk availability may also be involved in the beneficial effects of culling.

Consistent with previous findings [8], [9], [10], we found that, compared with SAL-treated rats, DEX-treated rats exhibited an increased basal CaMKII autophosphorylation at threonine-286 in hippocampal PSD fraction. It has been shown that threonine-286 phosphorylation is sufficient to convert the CaMKII to the high affinity form [35]. Thus, it is possible that the greater level of autophosphorylated CaMKIIα binds a greater amount of Ca^2+^/calmodulin, which in turn leads to systematic shift in favor of LTD at all submaximal levels of Ca^2+^ influx triggered by LFS. Indeed, it has been shown that transgenic mice overexpressing a mutated form of CaMKIIα with autonomous activity display a downward shift of the frequency response curve, favoring LTD over LTP induction in the hippocampal CA1 region [36]. Since the increase in CaMKIIα autophosphorylation observed in DEX-treated rats is correlated with a reduction of PP1 level, it is likely that neonatal DEX treatment may provide a unidentified mechanism for decreased PP1 production, which renders the CaMKIIα persistently hyperphosphorylated [9]. This notion is strengthened by our observation that culling reduced the lasting effects of neonatal DEX treatment on CaMKIIα and PP1 (Figure 6) and improved deficits in LTP and LTD. In addition, we observed that neonatal DEX treatment also led to a significant increase in the magnitude of DHPG-induced LTD and such effect was significantly reduced by litter culling (Figure 5). This finding is consistent with previous report showing that stress, through activation of glucocorticoid receptors by corticosterone, facilitates the induction of DHPG-induced LTD in hippocampal CA1 region [37]. However, it is not clear how neonatal DEX treatment exerted its long-lasting effects on mGluR-dependent LTD. Interestingly, a previous study [38] found that 17β-estradiol may facilitate the induction of DHPG-LTD in the hippocampal CA1 region through an enhancement of CaMKII activity. It is therefore possible that the lasting facilitatory effect of neonatal DEX treatment on DHPG-induced LTD is associated with a maintained increase in CaMKII activity. Additional studies are required to test this possibility.

With respect to long-lasting neurological outcome measures of neonatal DEX treatment, we have previously reported that this DEX regimen treatment may lead to an impairment of hippocampus-dependent contextual fear memory performance [9]. Besides affecting memory retention for one-trial passive avoidance task (Figure 8), the present study demonstrates that neonatal DEX treatment also elicited deficits in novel object recognition test (Figure 7). In rodents, the novel object recognition test has become the currently accepted standard for assessing recognition memory. Although novel object recognition probably involves a wide range of brain areas and neuronal processes, there is agreement that the hippocampus is critically important for normal recognition memory performance [39], [40]. The present results provide further support for this view by demonstrating that neonatal DEX treatment caused deficits in hippocampal synaptic plasticity and novel object recognition performance. Importantly, our data show that, in keeping with its effect on synaptic plasticity, culling effectively overcame the neonatal DEX treatment-induced impaired performance in hippocampus-dependent associative learning tasks.

In conclusion, our work reveals a critical role of growth retardation in lasting effects of neonatal DEX treatment on hippocampal synaptic plasticity and memory performance. Culling, perhaps due to greater maternal milk availability, is able to stimulate somatic growth and overcome synaptic and functional abnormalities resulting from neonatal DEX treatment. Although further investigations are needed to elucidate the molecular mechanisms involved in the culling effects, our findings demonstrate that supporting normal development and somatic growth may exert beneficial effects to reduce lasting adverse effects of neonatal DEX treatment on hippocampal function. These findings are of clinical importance because it is now difficult to avoid the use of corticosteroids in neonatology and perinatology to fight the problems of chronic lung disease.

## Materials and Methods

### Animals

Pregnant Sprague-Dawley rats (250–280 gm body weight) were housed in individual cages under the conditions of a 12 h light/dark cycle (lights on from 07:00 to 19:00 h) and *ad libitum* access to food and water. Pups were born on days 22–23 of gestation. On the day of birth (designated day 0), the number of pups per litter was culled to eight and four for Group A and B, respectively, with equal numbers of both sexes. Pups were weaned at 21 days of age and remained group housed with same-sex littermates until experimentation at 5 weeks of age. Only male rats were used in each experiment. All procedures were performed according to NIH guidelines for animal research (Guide for the Care & Use of Laboratory Animals, NRC, 1996) and were approved by the Institutional Animal Care and Use Committee of National Cheng Kung University (Approval No. 97225). All efforts were made to minimize animal suffering and to use only the number of animals necessary to produce reliable scientific data.

### Dexamethasone treatment

Four treatment groups were assigned for each litter: SAL- and DEX-treated pups from litters that had bee culled to 8 pups on P0; and SAL- and DEX-treated pups from litters that had bee culled to 4 pups on P0. All pups within each litter were removed from their home cage and separated from their mother for injection and body weight measurement (between 11:00 and 13:00 h) for a period of 5 min. Pups in the DEX-treated group received a daily intraperitoneal injection of DEX between P1 and P3. DEX was given in tapering doses of 0.5 mg/kg on P1, 0.3 mg/kg on P2, and 0.1 mg/kg on P3 as described previously [8], [9], [10]. Animals in the vehicle group received equivalent volumes of intraperitoneal injection of sterile SAL as the pups in the DEX-treated group. Electrophysiological, biochemical, immunohistochemical and behavioral experiments were performed using different groups of rats at the age of 5 weeks of age.

### Maternal care characterization

Behavioral observations were performed with mothers and their litters housed in 48×22×26 cm Plexiglas cages. Maternal care behaviors were video recorded using a digital video camera for six 60-min observation periods daily for the first 10 days postpartum and scoring was performed with the behavioral tracking system Ethovision (Noldus, Wageningen, The Netherlands) as described previously [19]. Pups were marked with nontoxic ink for identification. Observations were performed at four periods during the light phase (08:00, 11:00, 14:00 and 17:00 h) and two periods during the dark phase of the light/dark cycle (20:00 and 06:00 h). Within each observation period, the maternal behavior at a pup was scored every 3 min (20 observations per period ×6 periods per day  = 120 observations/pup/day). All observations were performed by individuals unaware of the origin of the pups. The following maternal behaviors were scored: (1) mother licking and grooming the pup, (2) mother nursing the pup in an arched-back posture, (3) mother nursing the pup in a flat-back posture, (4) passive posture in which the mother lays over the pup and (5) mother off the pup. The percentage of time spent in the maternal contact was analyzed.

### Hippocampal slice preparations and electrophysiology

Hippocampal slices (400 µm) were prepared from 5-week-old rats using standard procedures as described previously [9], allowed to recover for a minimum of 1 h, and then transferred to a submersion-type recording chamber continually perfused oxygenated artificial CSF (aCSF) solution (in mM: NaCl, 117; KCl, 4.7; CaCl_2_, 2.5; MgCl_2_, 1.2; NaHCO_3_, 25; NaH_2_PO_4_, 1.2; glucose, 11) at a flow rate of 2–3 ml/min at 32.0±0.5°**C**. Extracellular field potential recordings were carried out using an Axoclamp-2B amplifier (Axon Instruments, Union City, CA). Microelectrodes were pulled from microfibre 1.0 mm capillary tubing on a Brown-Flaming electrode puller (Sutter Instruments, San Rafael, CA). The responses were low pass filtered at 2 kHz, digitally sampled at 5–10 kHz, and analyzed using pCLAMP software (Version 7.0; Axon Instruments). Postsynaptic responses were evoked in CA1 stratum radiatum by stimulation of Schaffer collateral/commissural afferents at 0.033 Hz with a bipolar stimulating electrode. The stimulation strength was set to elicit response for which the amplitude was 30–40% of the maximum spike-free response. Field EPSPs were recorded with a glass pipette filled with 1 M NaCl (2–3 MΩ resistance) and the fEPSP slope was measured from approximately 20–70% of the rising phase using a least-squares regression. PPF ratio was assessed by using a succession of paired pulses separated by intervals of 20, 40, 60, 80, 150, and 200 ms. The LTP was induced by HFS, at the test pulse intensity, consisting of two 1-sec trains of stimuli separated by an intertrain interval of 20 sec at 100 Hz. The LTD was induced by LFS delivered at 1 Hz for 15 min (900 pulses) or a brief bath application of group I mGluR agonist DHPG (Tocris Cookson Ltd., Bristol, UK) for 5 min.

Whole-cell recordings were made from hippocampal CA1 pyramidal cells by using a patch clamp amplifier (Axopatch 200B, Axon Instruments) under infrared differential interference contrast optics. Electrical signals were low-pass filtered at 2 kHz, digitized at 10 kHz using a 12 bit analog-to-digital converter (Digidata 1320, Axon Instruments). An Intel Pentium-based computer with pCLAMP software (Version 8.0; Axon Instruments) and Mini Analysis 4.3 (Synaptosoft, Leonia, NJ) were used for on-line acquisition and off-line analysis of the data. For measurements of synaptically evoked IPSCs, a bipolar stainless steel stimulating electrode was placed in CA1 stratum radiatum to stimulate the Schaffer collateral/commissural afferents at 0.05 Hz and neurons were voltage-clamped at −70 mV in the presence of CNQX (20 µM) and D-APV (50 µM). Patch pipettes were pulled from borosilicate capillary tubing and heat polished. The electrode resistance was typically 3–6 MΩ. The composition of intracellular solution was (mM): CsCl, 150; HEPES, 10; MgCl_2_, 2; EGTA, 0.5; Na_2_ATP, 3; Na_3_GTP, 0.3 and sucrose to bring osmolarity to 290–295 mOsM and pH to 7.3. Miniature IPSCs were recorded at a holding potential of −70 mV in the presence of tetrodotoxin (TTX, 1 µM), CNQX (20 µM) and D-APV (50 µM). Detection threshold of 5 pA was used for mIPSCs. N-(2,6-dimethylphenyl)acetamide-2-triethylammonium bromide (QX-314, 5 mM) was included in the intracellular solution for IPSC and mIPSC recordings. For assessments of neuronal excitability, the whole-cell current-clamp was used to record spikes evoked by injection of depolarizing current pulses (0.2 nA, 500 ms) and CsCl in the intracellular solution was replaced with KCl. Series resistance and input resistance were monitored on-line throughout the recordings and data were discarded if resistance changed by more than 20%. The experiments were performed with the experimenter blind to the status of the animals from which slices were prepared.

### Passive avoidance training and testing

A one-way passive avoidance learning task was selected as the tool for behavioral assessment to measure associative memory retention performance in rats from each group as described previously [9]. Passive avoidance consisted of a training trial on the first day and a retention test 24 h later. On the training day, each rat was placed individually into the lit compartment of an automated passive avoidance system (Ugo Basile, Comerio, Italy) and, after entering the dark compartment, was given a scrambled foot shock (0.5 mA for 2 sec). Rats were tested for memory retention at 24 h after training. In the test section the foot shock was omitted; i.e., the conditioned stimulus (the apparatus) was not followed by unconditioned stimulus (electric shock). The latency to enter the dark compartment was scored. If a rat did not enter the chamber in 300 sec, the trial was terminated.

### Novel object recognition assay

The novel object recognition and memory retention test was used to examine recognition memory. The test apparatus consisted of a square chamber with ground area measuring 40×40 cm and object recognition test was conducted with dimmed light (approximately 10 Lux). The objects to be discriminated were a glass marble and a plastic dice. Before training, rats were habituated to the experimental arena by allowing them to freely explore it 10 min per day for 2 consecutive days in the absence of stimulus objects, and then returned to their home cages. In the training session, each rat was introduced for 5 min in the arena containing two identical objects (marble or dice). Locomotor activity and the duration for the rat spent in exploring the objects were video recorded using a digital video camera and scoring was performed with the behavioral tracking system Ethovision (Noldus, The Netherlands). Exploration was defined as sniffing or touching the stimulus object with the nose or forepaws. Sitting on or turning around the objects was not considered exploratory behavior. Twenty-four hours after training, rats were tested by reintroducing them in the arena for 5 min. One of the objects was familiar (presented during the training session) and the other was novel (never presented before). The amount of time spent exploring each object was recorded by an observer blind to the treatment. A discrimination index was calculated to determine preference of rats for exploration of the novel object relative to the familiar object in the test session. This index was calculated for each rat as proportion increase above the time each rat spent exploring the familiar object using the following formula: [(time exploring novel object − time exploring familiar object)/(time spend exploring familiar object + time spent exploring novel object)] ×100%.

### Preparation of postsynaptic density fractions

Isolation of PSD was performed according to the procedure of Wells et al [41]. Briefly, the microdissected CA1 regions were homogenized in ice-cold Ca^2+^, Mg^2+^-free buffer (50 mM HEPES, 100 mM NaCl, and 3 mM K acetate, pH 7.4) with RNase inhibitor (15 U/ml) and was centrifuged at 2000× g for 1 min. Supernatants were passed through two 100 µm nylon mesh filters, followed by a 5 µm pore filter. The filtrate was then centrifuged at 1000× g for 10 min and then was gently resuspended with same buffer at a protein concentration of 2 mg/ml.

### Western blotting

For each experimental group, homogenates from at least three slices were pooled. The microdissected subregions were lysed in ice-cold Tris-HCl buffer solution (TBS; pH 7.4) containing a cocktail of protein phosphatase and proteinase inhibitors (50 mM Tris-HCl, 100 mM NaCl, 15 mM sodium pyrophosphate, 50 mM sodium fluoride, 1 mM sodium orthovanadate, 5 mM EGTA, 5 mM EDTA, 1 mM phenylmethylsulfonyl fluoride, 1 µM microcystin-LR, 1 µM okadaic acid, 0.5% Triton X-100, 2 mM benzamidine, 60 µg/ml aprotinin, and 60 µg/ml leupeptin) to avoid dephosphorylation and degradation of proteins, and ground with a pellet pestle (Kontes glassware, Vineland, NJ, USA). Samples were sonicated and spun down at 15,000× g at 4°Cfor 10 min. The supernatant was then assayed for total protein concentration using Bio-Rad Bradford Protein Assay Kit (Hercules, CA). Each sample from tissue homogenate or PSD protein was separated in 10% SDS-PAGE gel. Following the transfer on nitrocellulose membranes, blots were blocked in buffer solution containing 5% milk and 0.1% Tween-20 in PBS (124 mM NaCl, 4 mM KCl, 10 mM Na_2_HPO_4_, and 10 mM KH_2_PO_4_, pH 7.2) for 1 h and then blotted for 2 h at room temperature with the monoclonal antibodies that recognizes phosphorylated CaMKIIα at threonine-286 site (1∶2000; Affinity Bioreagents, Golden, CO) or PP1 (1∶1000; Upstate Biotechnology, Lake Placid, NY, USA). It was then probed with HRP-conjugated secondary antibody for 1 h and developed using the ECL immunoblotting detection system (Amersham Biosciences, Buckinghamshire, UK), according to manufacturer's instructions. The immunoblots using phosphorylation site-specific antibodies were subsequently stripped and reprobed with an antibody that recognizes CaMKIIα (1∶1000; Affinity Bioreagents). The relative amount of CaMKIIα phosphorylation was analyzed by determining the ratio of the signals detected by using the phosphorylation site-specific antibody and the phosphorylation-independent antibody. Immunoblots were analyzed by densitometry using Bio-profil BioLight PC software. Only film exposures in the linear range of the ECL reaction were used for quantification analysis. Background correction values were subtracted from each lane to minimize variability across membranes.

### Histology and immunohistochemistry

Animals were deeply anesthetized with sodium pentobarbital (100 mg/kg, intraperitoneally) and perfused transcardially with 0.1 M PBS and 4% paraformaldehyde. After the perfusion, brains were removed and continue to fix in 4% paraformaldehyde for 48 h at 4°C and then transferred to the solution containing 30% sucrose that immersed in 4°C for at least 48 h before slicing. Coronal hippocampal slices (25 µm) were collected, washed with 0.3% Triton X-100, and then incubated for blocking with solution containing 3% goat serum in PBS. For quantitative evaluation of neuronal numbers with Nissl staining, sections were mounted directly on gelatin-coated glass slides and dried. The slides were stained with 1.0% cresyl violet, dehydrated through a series of ethanol, cleared, and coverslipped with permount (Fisher Scientific, Electron Microscopy Sciences, Washington, PA). For immunohistochemistry, the free-floating sections were washed with TBS-T wash buffer (10 mM Tris-HCl, 150 mM NaCl and 0.025% Tween 20; pH 7.4) and incubated with 1% H_2_O_2_ for 30 min to block endogenous peroxidase, and rinsed in PBS. The sections were incubated overnight at 4°C with a parvalbumin antibody (1∶1000; Millipore Bioscience Research Reagents, Temecula, CA) in PBS with 0.1% Triton X-100. Sections were then incubated with biotinylated secondary antibody (1∶200; Vector Laboratories, Burlingame, CA) for 90 min, followed by incubation with avidin and biotinylated peroxidase complex (Vector Laboratories). The immunostained sections were mounted on gelatin-subbed glass slides, dried, dehydrated, cleared, and coverslipped with permount. Specific staining was abolished by omission of primary antibody.

### Image acquisition and quantification

Nissl staining and parvalbumin immunostaining within hippocampal CA1 region were quantified in images from about 2.5 to 4.5 mm posterior to Bregma every sixth coronal section captured at 200× magnification and digitized with an Olympus BX51 microscope coupled to an Olympus DP70 digital camera (Olympus, Tokyo, Japan). Then, all images were imported into NIH ImageJ software for analysis. The number of interneurons labeled for parvalbumin in stratum origin and pyramidale of the CA1 region in neonatal saline- or dexamethasone-treated rats was estimated by using the optical fractionator-sampling method [42], [43]. A total of 4 counting frames (200×200 µm square) were sampled per CA1 region in each section. Neurons were counted only when the nucleus was in clear focus within the counting frame. The parvalbumin-immunoreactive cells in hippocampal CA1 region were classified into two distinct subpopulations: multi-polar round cells with a long axis: short axis ratio <1.5 and larger spindle-shaped cells with a long axis: short axis ratio ≥1.5 [44]. All counting was performed in a blind manner.

### Drugs

DHPG, CNQX, D-APV and TTX were purchased from Tocris Cookson (Bristol, UK); DEX was obtained from Sigma (St Louis, MO).

### Data analysis

All data are expressed as means ± SEM. Data of body weight, maternal care, stimulus-response curve of fEPSPs or IPSCs, PPF ratio, the average frequency or amplitude of mIPSCs, novel object recognition task and passive avoidance memory retention test were analyzed by one-way analysis of variance (ANOVA), followed by Fisher's least significance difference test when appropriate. For LTP and LTD experiments, statistical analysis was performed using the Mann-Whitney *U*-test. Western blot data were analyzed using unpaired Student's *t*-test. Number of animals used is indicated by n. Probability values of p<0.05 were considered to represent significant differences.

## Supporting Information

Figure S1 Effect of neonatal DEX treatment on the number of pyramidal neurons in hippocampal CA1 region. (A) Representative photographs with Cresyl violet staining of CA1 region showing that the number of pyramidal neurons was not significantly affected by neonatal DEX treatment compared with age-matched SAL-treated rats. (B) Group data showing the summary results from 4 rats of each group at 5 weeks of age.
(6.81 MB TIF)

Figure S2 Effect of neonatal DEX treatment on hippocampal CA1 neuronal excitability. Top, representative traces of action potential firing elicited by a constant depolarizing current injection (0.2 nA, 500 ms) in hippocampal CA1 pyramidal neurons from 5-week-old rats neonatally treated with SAL or DEX for Group A and Group B. The neurons were held at -70 mV in current-clamp mode. Bottom, summary of the number of action potentials by constant depolarizing current injection in slices from rats neonatally treated with SAL or DEX for Group A and Group B. The total number of animals examined is indicated by n in parenthesis.
(1.32 MB TIF)

Figure S3 Effect of neonatal DEX treatment on the number of parvalbumin (PA)-immunoreactive interneurons in the hippocampal CA1 region. (A) Representative photomicrographs with PV-immunostaining of CA1 region showing that the expression of PV-immunoreactive interneurons was not altered by neonatal DEX treatment in rats from Group A and Group B. (B) Group data showing the summary results from 6 rats of each group at 5 weeks old. The parvalbumin-immunoreactive cells in hippocampal CA1 region were classified into two distinct subpopulations: multi-polar round cells with a long axis: short axis ratio <1.5 and larger spindle-shaped cells with a long axis: short axis ratio > or  = 1.5.(n = 4 for each group).
(7.68 MB TIF)

Figure S4 Effect of neonatal DEX treatment on GABAA receptor-mediated inhibition of hippocampal CA1 pyramidal neurons. (A) Stimulus-response curve of IPSC amplitude versus stimulus intensity in the CA1 region of hippocampal slices from 5-week-old rats of Group A that received neonatal SAL or DEX treatment. Inset shows overlaid traces (each an average of 3 responses) evoked in a slice from a SAL- (left set of traces) and DEX-treated (right set of traces) rats. Monosynaptic IPSCs were evoked while holding neurons in voltage-clamp at -70 mV in the presence of CNQX (20 microM) and D-APV (50 microM). (B) Stimulus-response curve of IPSC amplitude versus stimulus intensity in the CA1 region of hippocampal slices from 5-week-old rats of Group B that received neonatal SAL or DEX treatment. (C) Representative traces and summary bar graph depicting the frequency and amplitude of mIPSCs of hippocampal CA1 pyramidal neurons from 5-week-old rats neonatally treated with SAL or DEX for Group A. Miniature IPSCs were recorded while holding neurons in voltage-clamp at -70 mV in the presence of CNQX (20 microM) and D-APV (50 microM). (D) Representative traces and summary bar graph depicting the frequency and amplitude of mIPSCs of hippocampal CA1 pyramidal neurons from 5-week-old rats neonatally treated with SAL or DEX for Group B. The total number of animals examined is indicated by n in parenthesis.
(2.29 MB TIF)
