# Supplementary figures and images for: The Role of Growth Retardation in Lasting Effects of Neonatal Dexamethasone Treatment on Hippocampal Synaptic Function
Source: PLoS One. 2010 Sep 21;5(9):e12806. doi: 10.1371/journal.pone.0012806 (PMC2943478; doi:10.1371/journal.pone.0012806)

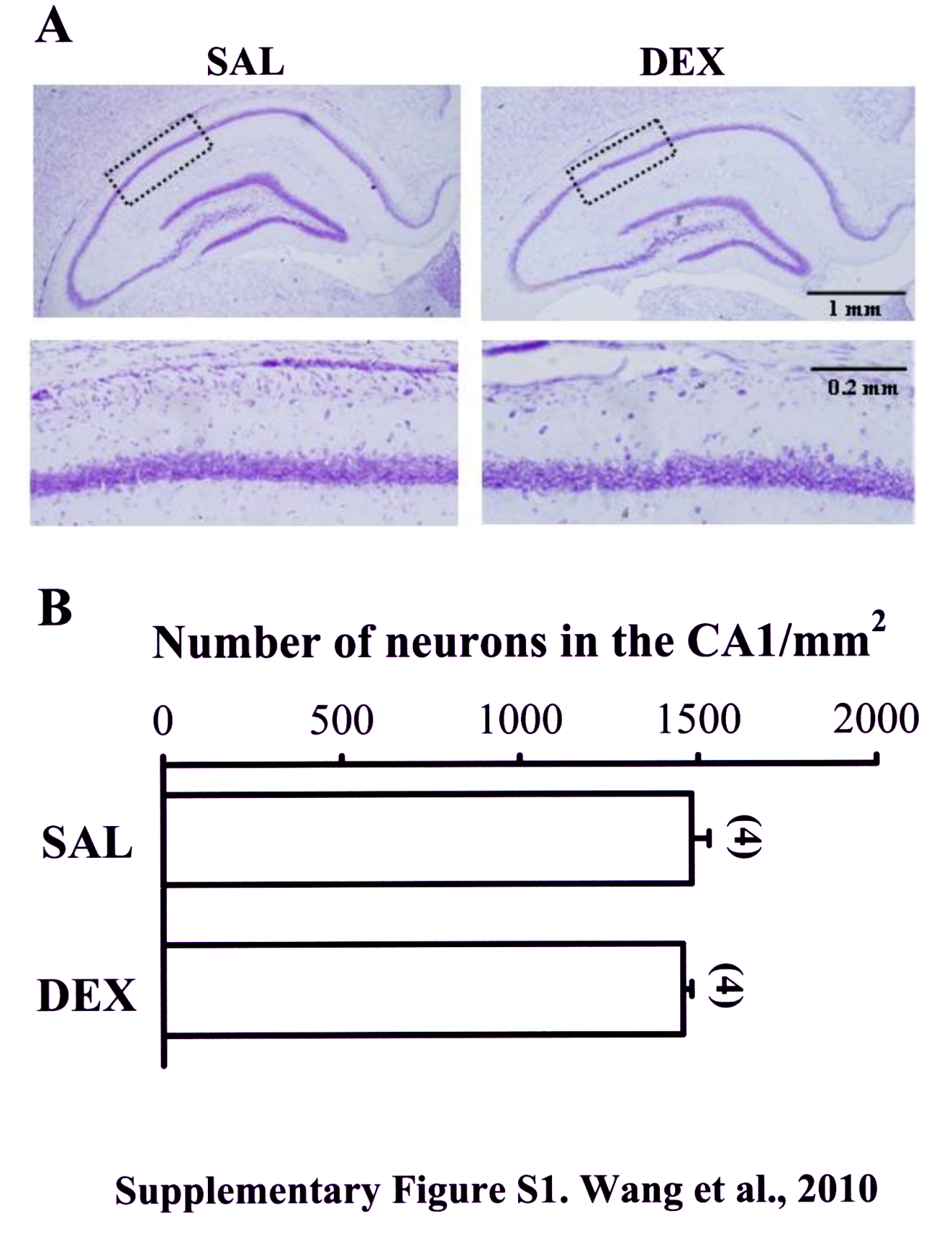

Supplement: Figure S1 — Effect of neonatal DEX treatment on the number of pyramidal neurons in hippocampal CA1 region. (A) Representative photographs with Cresyl violet staining of CA1 region showing that the number of pyramidal neurons was not significantly affected by neonatal DEX treatment compared with age-matched SAL-treated rats. (B) Group data showing the summary results from 4 rats of each group at 5 weeks of age. (6.81 MB TIF) [file pone.0012806.s001.tif]

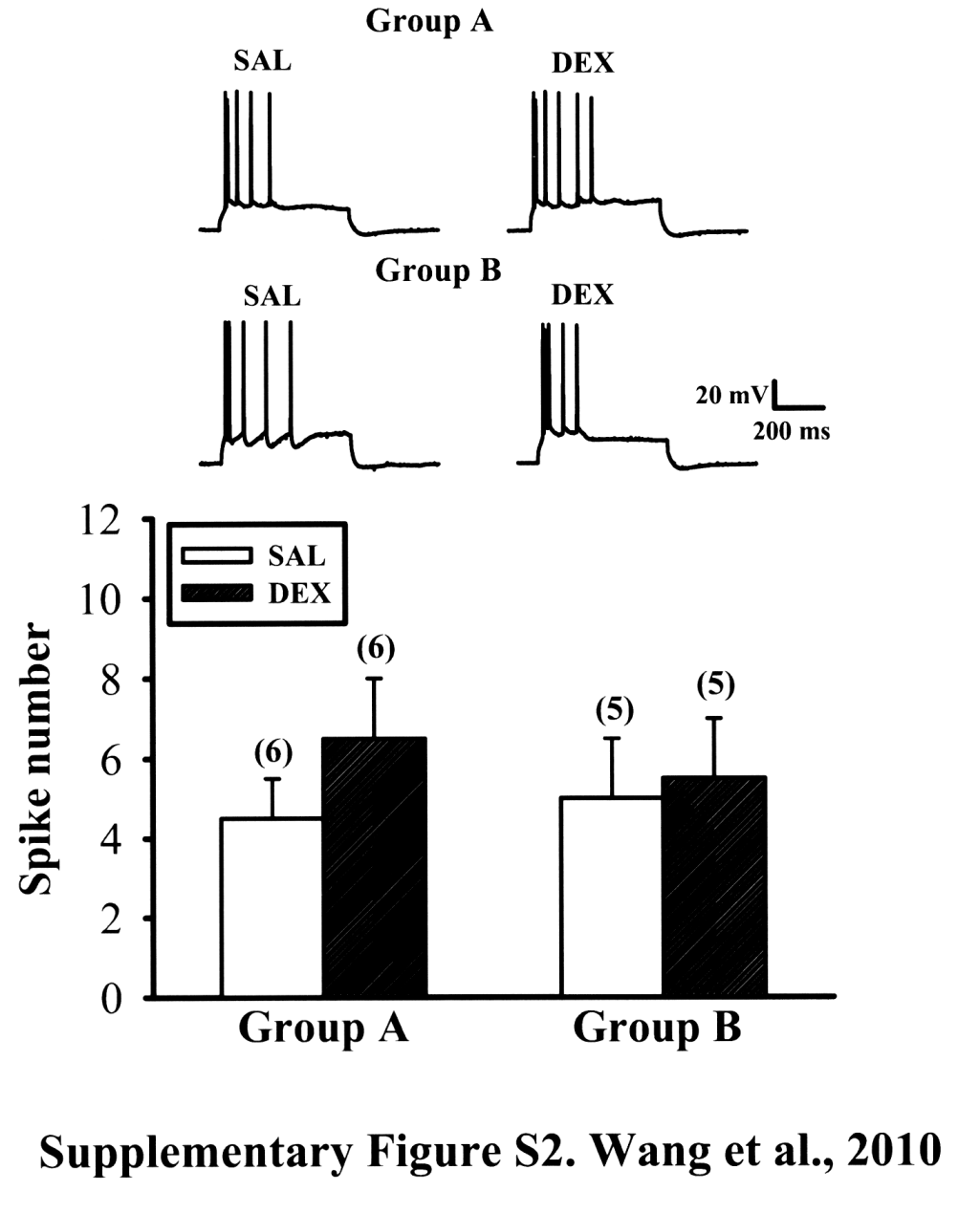

Supplement: Figure S2 — Effect of neonatal DEX treatment on hippocampal CA1 neuronal excitability. Top, representative traces of action potential firing elicited by a constant depolarizing current injection (0.2 nA, 500 ms) in hippocampal CA1 pyramidal neurons from 5-week-old rats neonatally treated with SAL or DEX for Group A and Group B. The neurons were held at -70 mV in current-clamp mode. Bottom, summary of the number of action potentials by constant depolarizing current injection in slices from rats neonatally treated with SAL or DEX for Group A and Group B. The total number of animals examined is indicated by n in parenthesis. (1.32 MB TIF) [file pone.0012806.s002.tif]

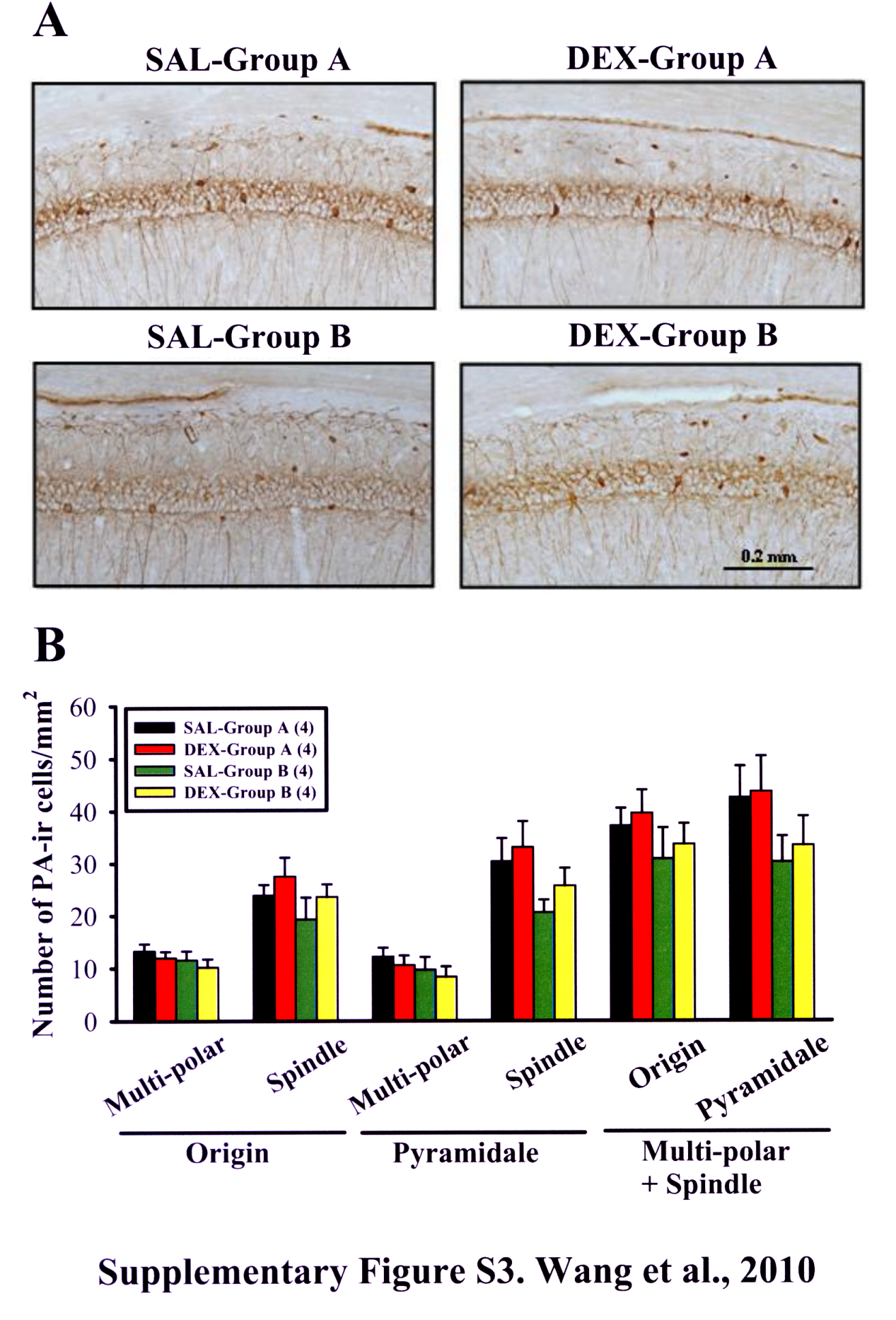

Supplement: Figure S3 — Effect of neonatal DEX treatment on the number of parvalbumin (PA)-immunoreactive interneurons in the hippocampal CA1 region. (A) Representative photomicrographs with PV-immunostaining of CA1 region showing that the expression of PV-immunoreactive interneurons was not altered by neonatal DEX treatment in rats from Group A and Group B. (B) Group data showing the summary results from 6 rats of each group at 5 weeks old. The parvalbumin-immunoreactive cells in hippocampal CA1 region were classified into two distinct subpopulations: multi-polar round cells with a long axis: short axis ratio <1.5 and larger spindle-shaped cells with a long axis: short axis ratio > or = 1.5.(n = 4 for each group). (7.68 MB TIF) [file pone.0012806.s003.tif]

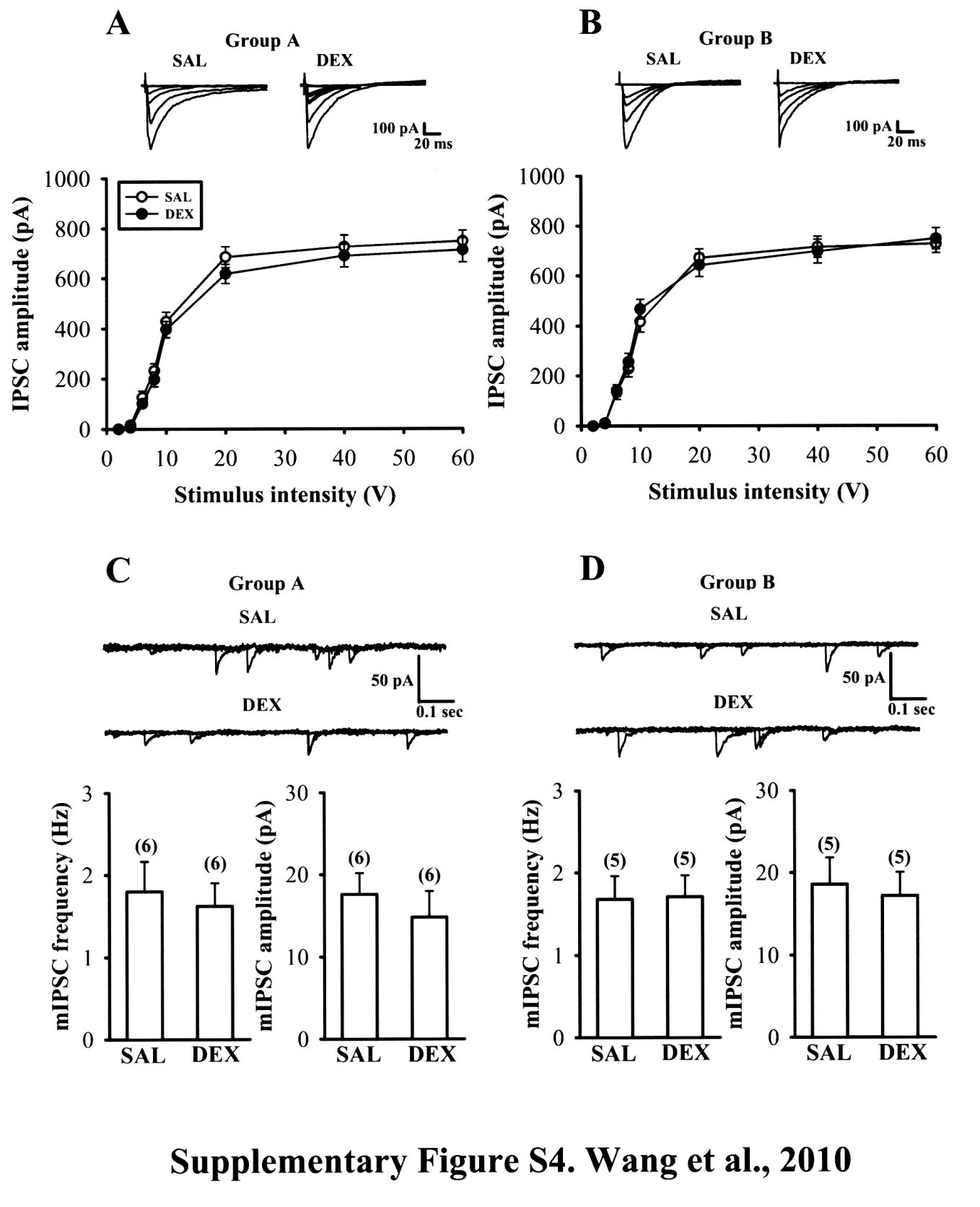

Supplement: Figure S4 — Effect of neonatal DEX treatment on GABAA receptor-mediated inhibition of hippocampal CA1 pyramidal neurons. (A) Stimulus-response curve of IPSC amplitude versus stimulus intensity in the CA1 region of hippocampal slices from 5-week-old rats of Group A that received neonatal SAL or DEX treatment. Inset shows overlaid traces (each an average of 3 responses) evoked in a slice from a SAL- (left set of traces) and DEX-treated (right set of traces) rats. Monosynaptic IPSCs were evoked while holding neurons in voltage-clamp at -70 mV in the presence of CNQX (20 microM) and D-APV (50 microM). (B) Stimulus-response curve of IPSC amplitude versus stimulus intensity in the CA1 region of hippocampal slices from 5-week-old rats of Group B that received neonatal SAL or DEX treatment. (C) Representative traces and summary bar graph depicting the frequency and amplitude of mIPSCs of hippocampal CA1 pyramidal neurons from 5-week-old rats neonatally treated with SAL or DEX for Group A. Miniature IPSCs were recorded while holding neurons in voltage-clamp at -70 mV in the presence of CNQX (20 microM) and D-APV (50 microM). (D) Representative traces and summary bar graph depicting the frequency and amplitude of mIPSCs of hippocampal CA1 pyramidal neurons from 5-week-old rats neonatally treated with SAL or DEX for Group B. The total number of animals examined is indicated by n in parenthesis. (2.29 MB TIF) [file pone.0012806.s004.tif]
